# Long-term safety and stability of angiogenesis induced by balanced single-vector co-expression of PDGF-BB and VEGF_164_ in skeletal muscle

**DOI:** 10.1038/srep21546

**Published:** 2016-02-17

**Authors:** Roberto Gianni-Barrera, Maximilian Burger, Thomas Wolff, Michael Heberer, Dirk J. Schaefer, Lorenz Gürke, Edin Mujagic, Andrea Banfi

**Affiliations:** 1 Cell and Gene Therapy, Department of Biomedicine, Basel University, Basel, Switzerland; 2 Department of Surgery, Basel University Hospital, Basel, Switzerland; 3 Plastic, Reconstructive, Aesthetic and Hand Surgery, Basel University Hospital, Basel, Switzerland; 4 Vascular Surgery, Basel University Hospital, Basel, Switzerland

## Abstract

Therapeutic angiogenesis by growth factor delivery is an attractive treatment strategy for ischemic diseases, yet clinical efficacy has been elusive. The angiogenic master regulator VEGF-A can induce aberrant angiogenesis if expressed above a threshold level. Since VEGF remains localized in the matrix around expressing cells, homogeneous dose distribution in target tissues is required, which is challenging. We found that co-expression of the pericyte-recruiting factor PDGF-BB at a fixed ratio with VEGF from a single bicistronic vector ensured normal angiogenesis despite heterogeneous high VEGF levels. Taking advantage of a highly controlled gene delivery platform, based on monoclonal populations of transduced myoblasts, in which every cell stably produces the same amount of each factor, here we rigorously investigated a) the dose-dependent effects, and b) the long-term safety and stability of VEGF and PDGF-BB co-expression in skeletal muscle. PDGF-BB co-expression did not affect the normal angiogenesis by low and medium VEGF doses, but specifically prevented vascular tumors by high VEGF, yielding instead normal and mature capillary networks, accompanied by robust arteriole formation. Induced angiogenesis persisted unchanged up to 4 months, while no tumors appeared. Therefore, PDGF-BB co-expression is an attractive strategy to improve safety and efficacy of therapeutic angiogenesis by VEGF gene delivery.

Coronary and peripheral artery diseases are still a major cause of morbidity and mortality in Western countries despite optimal medical and surgical treatment^1^. Therapeutic angiogenesis, i.e. the growth of new blood vessels by the delivery of specific growth factors in order to restore the perfusion of tissue distal to a vascular occlusion, is an attractive strategy to fill this unmet clinical need. Vascular endothelial growth factor-A (VEGF) is the master regulator of vascular growth in both physiological and pathological conditions^2^. However, it has been shown that the uncontrolled delivery of VEGF to both ischemic and non-ischemic tissues by a variety of methods causes excessive vascular proliferation, with the growth of aberrant vessels and angioma-like vascular tumors^3^,^4^,^5^,^6^,^7^,^8^. Since VEGF_164_ remains tightly bound to the extracellular matrix^9^, the induction of normal or aberrant angiogenesis has been found to depend on the concentration of VEGF in the microenvironment around each producing cell *in vivo*, rather than its total dose, both in normal and ischemic muscle^10^,^11^. When microenvironmental concentrations are homogeneously distributed in the tissue, a range of doses yields only morphologically normal capillaries, whereas angioma-like vascular structures are induced above a distinct threshold level^10^. However we recently found that the transition between normal and aberrant angiogenesis does not depend exclusively on VEGF dose, but rather on the balance between endothelial stimulation by VEGF and pericyte recruitment by Platelet-derived growth factor-BB (PDGF-BB)^12^. In fact, when endogenous PDGF-BB signaling was blocked, even low VEGF levels caused angioma-like vascular structures and, conversely, balanced co-expression of both factors from a single bicistronic vector prevented aberrant angiogenesis by uncontrolled and high VEGF levels, yielding instead only mature and morphologically normal capillary networks after 2 weeks, which significantly improved blood flow and collateral growth in a mouse model of hindlimb ischemia^12^. In agreement with these findings, VEGF and PDGF-BB co-delivery by AAV vectors significantly improved the functional efficacy of VEGF alone in ischemic models both in rabbit hindlimb and pig myocardium, while enabling a 5-fold vector dose reduction^13^. Also, sequential release of the two recombinant factors from hydrogels in ischemic mouse myocardium could increase the induction of mature arteriole-like vessels, without affecting capillary growth, leading to improved cardiac function^14^.

Based on these results, balanced co-expression of VEGF and PDGF-BB is a promising strategy to overcome the limitations of VEGF gene delivery in pro-angiogenic therapeutic approaches. However, it is currently unknown: 1) whether and how PDGF-BB co-expression may modify the angiogenic responses induced by specific VEGF doses; and 2) the safety of long-term co-expression of VEGF and PDGF-BB in a therapeutically relevant range of doses. A major hurdle has been the paucity of tools available to precisely control the amount of both factors in the microenvironment around each producing cell *in vivo*. Therefore, here we took advantage of a highly controlled platform for sustained gene expression in skeletal muscle that we developed in the last decade^10^,^15^, based on monoclonal populations of transduced myoblasts, in which every cell stably produces a specific amount of VEGF alone or at a fixed ratio with PDGF-BB, to rigorously investigate a) the dose-dependent effects, and b) the long-term safety and stability of angiogenesis induced by balanced and constitutive co-expression of VEGF and PDGF-BB in the therapeutic target tissue of skeletal muscle.

## Results

### Generation, *in-vitro* characterization and selection of V, P and VIP myoblast clones

**Figure 1:**
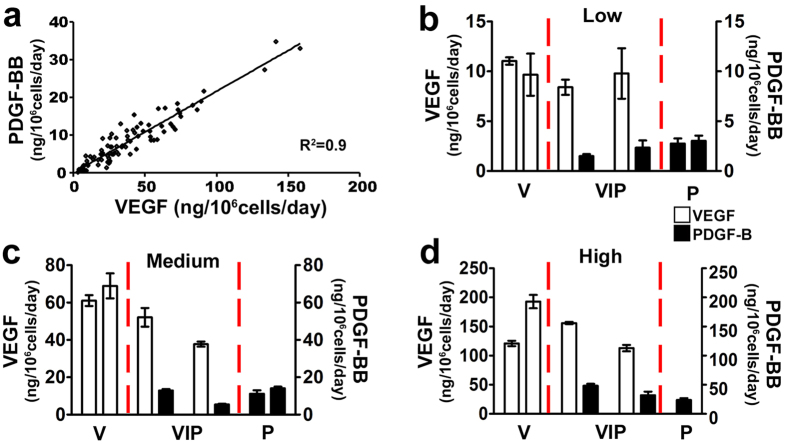
Balanced VEGF and PDGF-BB expression by VIP myoblast clones and selection of clone families. (**a**) Correlation between *in vitro* production of VEGF_164_ and PDGF-BB (in ng/10^6^ cells/day) in 90 individual myoblast VIP clones, co-expressing the two factors from a single bicistronic retroviral vector. The relative levels were linearly correlated in each clone (R^2^ = 0.9). (**b–d**) Based on their *in vitro* production of VEGF and/or PDGF-BB, 6 VIP clones were paired to 6 V clones and 5 P clones, expressing only VEGF and PDGF-BB respectively, in 3 different groups of increasing expression levels (Low, Medium and High). Data are shown as mean ± SEM (n = 4/clone).

Primary mouse myoblasts were transduced with a bicistronic retroviral vector (named pAMFG-VIP for *Vegf*-IRES-*Pdgfb*) expressing murine VEGF_164_ and human PDGF-BB at a fixed relative ratio, as previously described^12^. To rigorously study the dose-dependent effects of PDGF-BB co-expression on VEGF-induced angiogenesis, a pool of 90 monoclonal populations was isolated from the VIP polyclonal myoblasts (VIP clones). VEGF and PDGF-BB expression were measured by ELISA in all clones and they were found to cover a wide range of levels (VEGF: 3.0 ± 0.3 to 158.5 ± 2.3 ng/10^6^ cells/day; PDGF-BB: 0.2 ± 0.0 to 33.0 ± 0.4 ng/10^6^ cells/day). The ratio of PDGF-BB:VEGF_164_ production in individual clones was of 0.36 ± 0.02 on a molar basis with a very high correlation coefficient of R^2^ = 0.9 (Fig. 1a), showing that the two factors were produced at a fixed ratio in each individual cell, regardless of the absolute amounts. A similar pool of 20 monoclonal populations expressing only PDGF-BB (P clones) was isolated from myoblasts transduced with a retrovirus carrying the *Pdgfb* sequence alone. The PDGF-BB production of P clones ranged from 0.8 ± 0.03 to 28.7 ± 1.3 ng/10^6^cells/day, covering the same spectrum as the VIP clones. A previously generated library of 21 VEGF-expressing monoclonal populations^10^,^11^,^15^ produced only murine VEGF_164_ in a range between 0.8 ± 0.1 and 191.2 ± 13.5 ng/10^6^cells/day (V clones), also covering a similar spectrum as the VIP clones. Based on their *in vitro* production of VEGF, three groups of two VIP clones each were selected: VIP-low (VEGF range: 5–15 ng/10^6^ cells/day), VIP-medium (VEGF range: 30–70 ng/10^6^ cells/day) and VIP-high (VEGF range: > 100 ng/10^6^ cells/day) (Fig. 1b–d). Each group was paired with two V and two P clones, selected from the libraries described above and secreting equivalent amounts of either VEGF or PDGF-BB alone (except for the high PDGF-BB group, where only one clone was available) (Fig. 1b–d). All retroviral vectors also expressed a truncated version of CD8a as a convenient FACS-sortable cell surface marker^15^ and cells transduced with the empty retroviral vector expressing only CD8a (control cells) were used as negative control.

### PDGF-BB co-expression prevents aberrant angiogenesis by high VEGF levels, but does not affect vessel quantity nor size by low and medium VEGF

**Figure 2:**
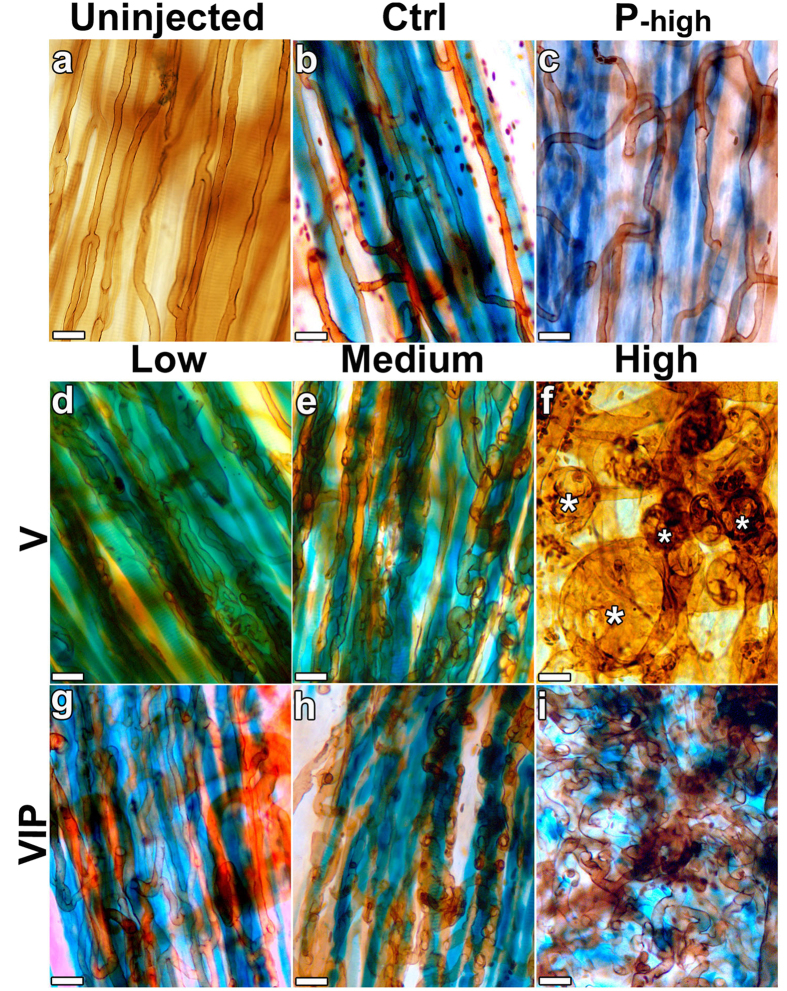
PDGF-BB co-expression switches aberrant angiogenesis by high VEGF doses to normal. (**a–i**) Control myoblasts (Ctrl) or myoblast clones expressing VEGF (V), PDGF-BB (P) or both (VIP) at low, medium or high levels were implanted into the ear muscles of SCID mice. Four weeks later, vascular morphology was analyzed in tissue whole-mounts by intravascular lectin staining (brown) and myoblast engraftment was revealed by X-Gal staining (blue; n = 5–10 samples/group). Asterisks indicate angioma-like aberrant vascular structures. Size bars = 20 μm.

To determine whether and how PDGF-BB co-expression modified the effects of specific microenvironmental VEGF doses, vascular morphology was evaluated 4 weeks after implantation of the selected VIP, V and P clones in the ear muscle (auricularis posterior) of SCID mice. The thin and accessible muscle layer in the dorsal aspect of the external mouse ear is ideally suited to whole-mount analysis of morphological changes in vascular networks: myoblast engraftment was detected by X-gal staining and vascular morphology was visualized by intravascular lectin staining, as previously established^10^. Control CD8 cells and all P clones (represented here for convenience only by the highest PDGF-BB producer, P-high) did not alter the pre-existing vasculature, made up mostly of homogeneous capillaries running parallel to the muscle fibers (Fig. 2a–c; n = 6 control cells, n = 5–10 for each P clone). As expected^10^,^15^, low and medium V clones induced only normal angiogenesis, made up of homogeneous capillary-sized microvessels, whereas V-high clones caused the appearance of large angioma-like vascular structures, characterized by the bulb-like and progressive circumferential enlargement of discrete vessel segments of heterogeneous sizes (Fig. 2d–f; n = 8). All the VIP clones, which belonged to the specific groups of VEGF expression, induced vessels of morphology that did not differ between each other. PDGF-BB co-expression did not qualitatively affect the normal angiogenesis induced by low and medium VEGF levels (VIP-low and VIP-med clones), yielding normal capillaries that wrapped around each single transduced muscle fiber (Fig. 2g,h; n = 8). Remarkably, the VIP-high clones induced only dense networks of morphologically normal capillaries, with no instances of angioma-like structures, despite high levels of VEGF expression (Fig. 2i; n = 10).

**Figure 3:**
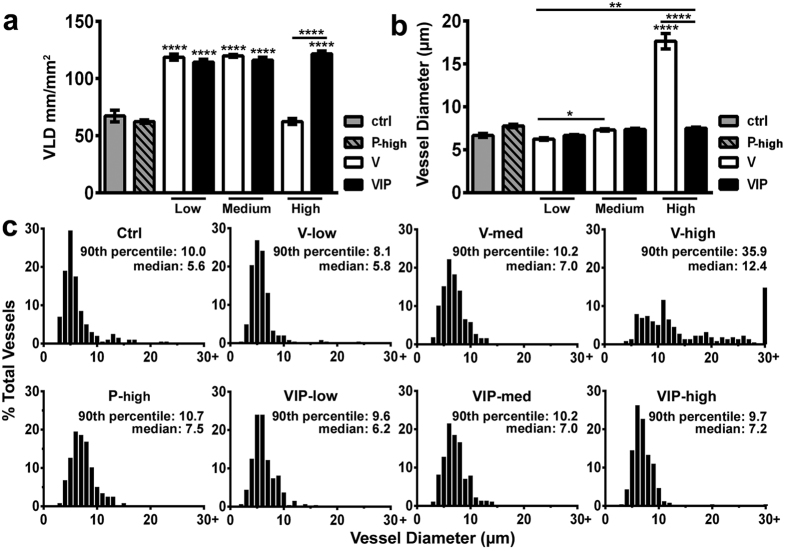
PDGF-BB co-expression does not affect normal angiogenesis by low and medium doses of VEGF alone. (**a**) The amount of angiogenesis was quantified by measuring vessel length density in areas of myoblast engraftment on tissue whole-mounts after intravascular lectin staining (n = 10–18 measurements/group in 5–10 tissues/group). (**b,c**) Vessel diameters (in μm) were quantified in the same areas (n > 200/group in 5–10 tissues/group) and results are shown as mean ± SEM (**b**) and as the distribution of vessel diameters over 1-μm intervals (**c**) VLD = vessel length density (mm of vessel length/mm^2^ of area of effect); *p < 0.05, **p < 0.01 and ****p < 0.0001 (ANOVA with Sidak multiple comparisons test).

The amount of induced vascularity was quantified by measuring the vessel length density (VLD), corresponding to the total vascular length in a given area independent of vessel size or morphology (Fig. 3a). All PDGF-BB levels (P-low, P-med and P-high clones) did not induce any angiogenesis and VLD in the implantation areas did not increase compared to control cells, as expected (62.5 ± 1.5 mm/mm^2^, 63.1 ± 2.3 mm/mm^2^ and 62.5 ± 1.5 mm/mm^2^, respectively; control cells = 67.3 ± 5.1 mm/mm^2^). Since the results were similar between the three PDGF-BB doses, only the VLD of the P-high condition is shown in Fig. 3a. Low and medium V clones induced a marked increase in VLD compared to control cells (118.7 ± 2.7 and 119.9 ± 1.2 mm/mm^2^, respectively; p < 0.0001), but PDGF-BB co-expression did not further increase the amount of angiogenesis (VIP-low = 114.4 ± 2.5, VIP-med = 116.2 ± 2.3 mm/mm^2^, p = N.S. vs the corresponding V clones). On the other hand, high VEGF levels did not increase VLD compared to controls (62.5 ± 2.6 mm/mm^2^, p = N.S.), because the induced aberrant angiogenesis comprised almost exclusively circumferentially enlarged bulbous vascular segments, which did not contribute significant increases to the vascular length of the network (Fig. 2f). However, PDGF-BB co-expression with high VEGF levels significantly increased VLD compared with VEGF alone (VIP-high = 121.7 ± 2.5 mm/mm^2^ vs V-high = 62.5 ± 2.6 mm/mm^2^, p < 0.0001), restoring it to values similar to those obtained with low and medium VEGF levels, as the angioma-like vascular structures were replaced by normal capillary networks (Fig. 2i).

Further, vessel diameters were measured on randomly selected fields using a standardized grid overlay and were shown as average values ± SEM (Fig. 3b) or size distributions (Fig. 3c). Pre-existing normal capillaries in areas implanted with control CD8 cells had homogeneous sizes, tightly distributed around a median of 5.6 μm and with a 90^th^ percentile value of 10 μm. Surprisingly PDGF-BB induced a moderate enlargement of the pre-existing capillaries, with an apparent dose-dependent trend, which however was not significant (median of 6.8 μm, 7.2 μm and of 7.5 μm for P-low, P-med and P-high, respectively; only the P-high condition is shown in Fig. 3b,c). New vessels induced by V-low and V-med clones had homogeneous capillary-like size distributions, but a VEGF dose-dependent increase in diameters was observed (median = 5.8 μm and 7 μm for V-low and V-med, respectively; p < 0.05 in Fig. 3b), as previously described^11^, whereas high levels of VEGF alone generated vascular structures with very heterogeneous diameters, 20.4% of which were larger than 15 μm (90^th^ percentile = 35.9 μm; p < 0.0001 in Fig. 3b). PDGF-BB co-expression did not significantly change the size of vessels induced by equivalent doses of VEGF alone in the range inducing normal angiogenesis, despite a slight increase in the median value of diameter distributions (6.2 μm and 7.5 μm for VIP-low and VIP-medium, respectively). The completely normal angiogenesis induced by PDGF-BB co-expression with high VEGF levels (VIP-high) was comprised of homogeneous capillary-size micro-vascular networks (90^th^ percentile = 9.7 μm with only 0.8% of vessels larger than 15 μm). Interestingly, the diameters of these normalized vessels were not further increased compared to the V-med/VIP-med conditions (median = 7.2 μm; p = N.S. in Fig. 3b), suggesting that the VEGF dose-dependent increase in the size of new vessels is limited to a maximum value compatible with normal angiogenesis.

### Vascular maturation

**Figure 4:**
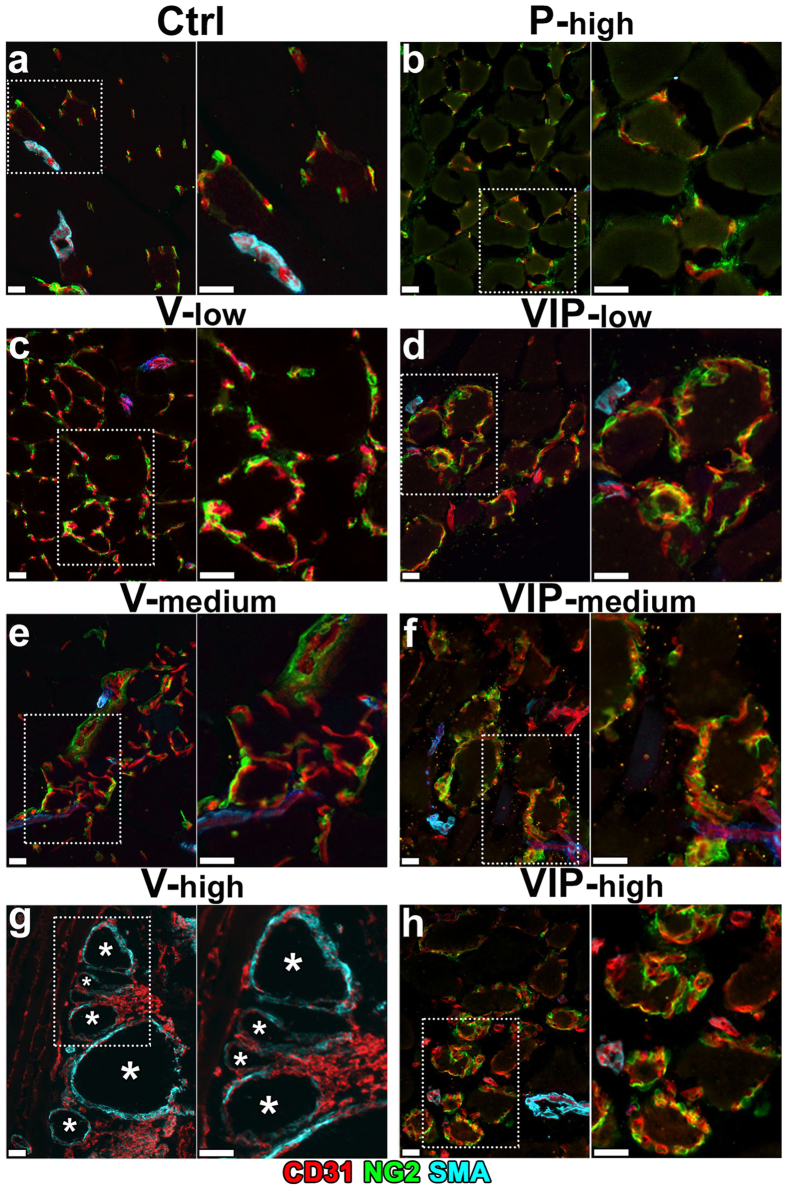
PDGF-BB co-expression restores normal pericyte coverage of vessels induced by high VEGF. V, VIP and P myoblast clones, expressing low, medium or high levels of one or both factors, as well as control cells (Ctrl), were implanted into calf muscles of SCID mice. Frozen sections were obtained 4 weeks after implantation and immunostained for CD31 (endothelium, in red), NG2 (pericytes, in green) and α-SMA (smooth muscle cells, in cyan); n = 3–8 samples/group. In each panel, the right image is a higher-magnification view of the area marked by the white dotted box in the left image. Size bars = 20 μm; asterisks indicate angioma-like aberrant vascular structures.

Vascular maturation was investigated by assessing pericyte coverage of the induced endothelial structures. Pre-existing capillaries in muscles treated with control cells and all PDGF-expressing groups (P-low, P-med and P-high), in which no angiogenesis was induced, were uniformly covered with typical pericytes, staining positive for nerve/glial antigen 2 (NG2) and negative for α-SMA (Fig. 4a,b; n = 6 for control cells and n = 2–3 for each P group). Remarkably, in all muscles implanted with P clones NG2-positive pericytes could only be observed in close contact with endothelial structures, but not in the interstitial spaces among the myofibers, showing that sustained PDGF-BB expression did not lead to excessive pericyte proliferation. As previously described, newly induced capillaries by both low and medium VEGF were also invested by normal NG2-positive pericytes (Fig. 4c,e), whereas high VEGF caused the appearance of large angioma-like aberrant vascular structures, devoid of pericytes and associated with a typical coverage of NG2-negative and α-SMA-positive smooth muscle cells (Fig. 4g). PDGF-BB co-expression with low or medium VEGF levels (VIP-low and VIP-med) not only did not affect the maturation of the normal capillaries that were induced, which were uniformly covered with NG2-positive pericytes, but also did not cause proliferation and accumulation of additional NG2-positive cells in the interstitial spaces between muscle fibers at either level of expression (Fig. 4d,f). In agreement with the results in the ear muscle (Fig. 2), PDGF-BB co-expression caused complete morphological normalization of the vasculature induced by high VEGF levels (VIP-high) in limb muscles as well. Such normalized capillary networks displayed a physiological association with NG2-positive pericytes instead of the thick smooth muscle coating of angioma-like structures generated by the same doses of VEGF alone. Again, no additional NG2-positive or α-SMA-positive cells were found outside of vessel walls in the interstitial spaces between muscle fibers (Fig. 4h).

### *In vivo* expression kinetics of delivered and endogenous VEGF and PDGF-BB

**Figure 5:**
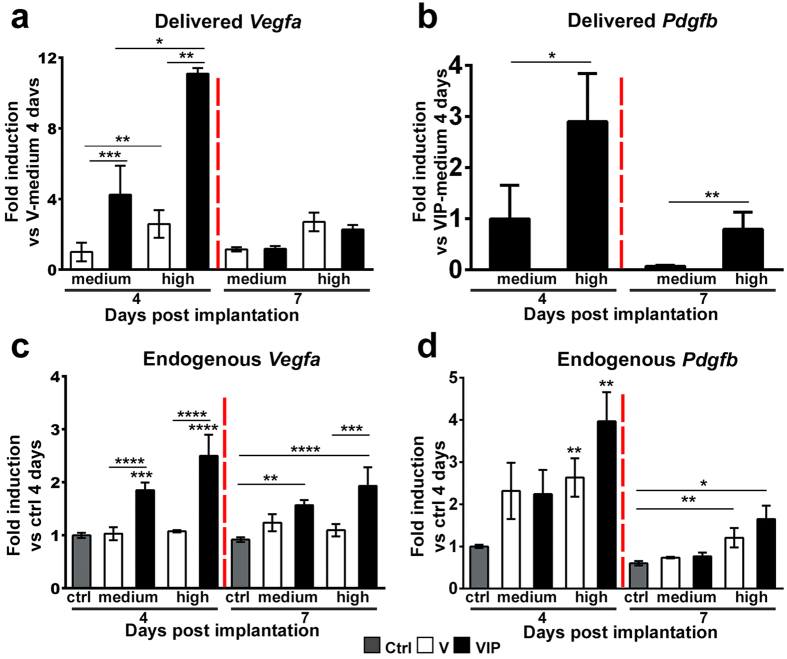
*In vivo* expression of delivered and endogenous factors. *In vivo* expression of the *Vegfa* and *Pdgfb* genes, discriminating between the delivered and endogenous transcripts, was quantified in calf muscles 4 and 7 days after implantation with control cells (Ctrl) and myoblast clones expressing medium or high levels of VEGF alone or with PDGF-BB (V and VIP, respectively). Data are shown as mean ± SEM (n = 4 muscles/condition); *p < 0.05, **p < 0.01, ***p < 0.001 and ****p < 0.0001 (ANOVA with Sidak multiple comparisons test after data normalization by logarithmic transformation).

The specific monoclonal populations of transduced myoblasts were selected on the basis of their transgene expression levels measured *in vitro*. However, if VEGF expression by the VIP clones were lost *in vivo* more than that by the equivalent V clones, particularly during the crucial initial stages of vascular induction, this would lead to a lower effective VEGF production in the tissue and could potentially explain the lack of aberrant angiogenesis even without a functional effect of PDGF-BB. Therefore, we sought to verify this possibility by quantifying *in vivo* gene expression by qRT-PCR in calf muscles four and seven days after implantation of V and VIP clones expressing medium and high VEGF levels, as the switch between normal and aberrant angiogenesis, as well as its reversal by PDGF-BB co-expression, takes place between these doses. These time-points were chosen because we previously found that the morphogenic events leading to either normal or aberrant structures by increasing VEGF doses take place between four and seven days after implantation, while no new vascular growth takes place afterwards^16^. Gene expression analysis with specific primers for the viral vector-encoded factors confirmed that *in vivo* expression of *Vegfa* from VIP clones was similar or higher than that of equivalent V clones at both time-points (Fig. 5a). As expected, expression of delivered human *Pdgfb* also remained higher with the VIP-high clone compared to the VIP-med clone at both time points (Fig. 5b). Expression of the endogenous *Vegfa* and *Pdgfb* genes was also quantified, using specific primers spanning the 5′-UTR sequence of the murine *Vegfa* mRNA, which is absent in the retroviral cassette, and a commercial assay specific for murine *Pdgfb*, respectively. Endogenous *Vegfa* was not affected by V clones, but was up-regulated by both VIP clones at both four and seven days (Fig. 5c). Endogenous *Pdgfb* was up-regulated by all clones at four days and expression declined by seven days, consistently with the transient activation of endothelium during the initial stage of angiogenesis. However, it remained similarly and slightly elevated compared to baseline in both V-high and VIP-high conditions (Fig. 5d).

### Long-term persistence and vascular maturation

**Figure 6:**
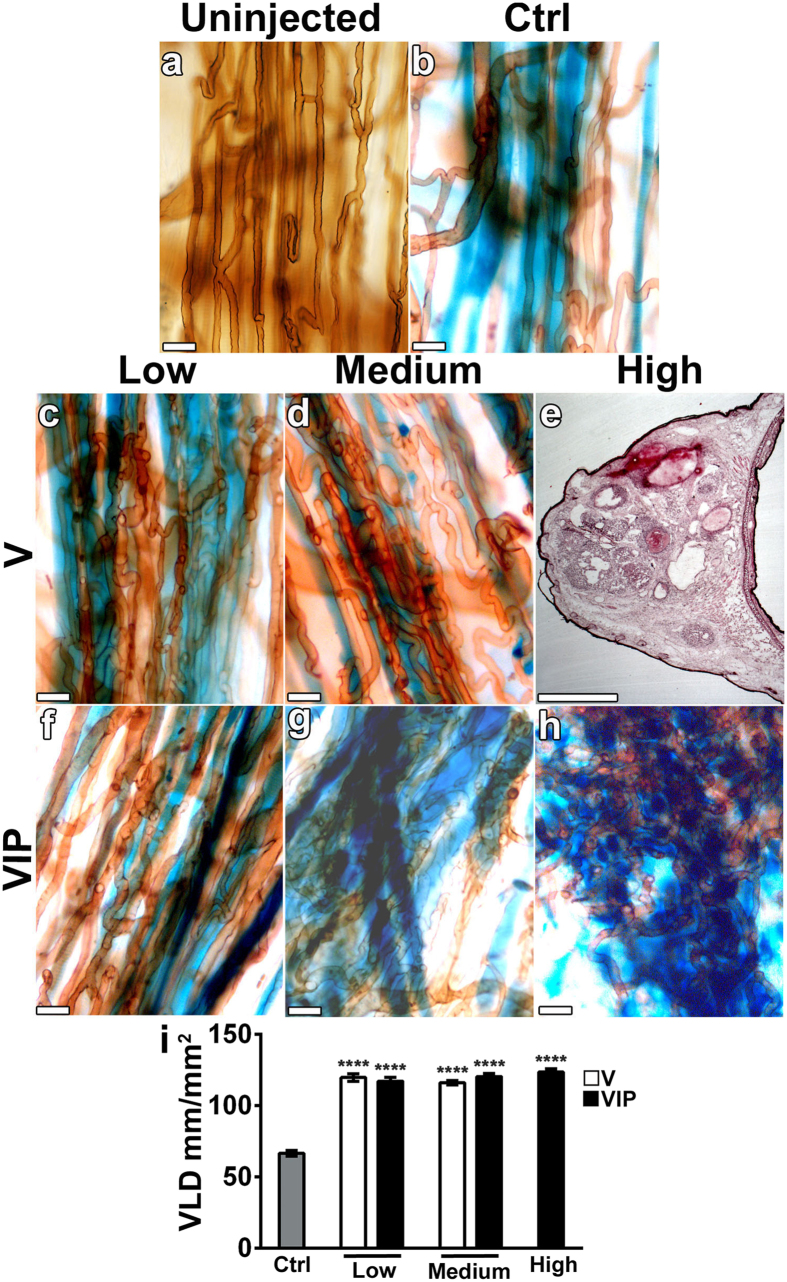
Normal vasculature induced by VEGF and PDGF-BB co-expression persists unchanged up to 4 months. (**a–h**) Control cells (Ctrl) or V and VIP myoblast clones expressing low, medium or high levels were implanted into the ear muscles of SCID mice. Four months later, vascular morphology was analyzed in tissue whole-mounts by intravascular lectin staining (brown) and myoblast engraftment was revealed by X-Gal staining (blue; n = 5–9/group). High VEGF levels caused the progressive growth of macroscopic angiomas (**e**) and required early termination of the experiment after 6 weeks (n = 4). Size bars = 20 μm, except in (**e**): size bar = 1mm. (**i**) The amount of angiogenesis was quantified by measuring vessel length density (VLD) in areas of myoblast engraftment. Data are shown as mean  ±  SEM (n = 9–18 measurements/group in 5–9 tissues/group); ****p < 0.0001 vs control (ANOVA with Sidak multiple comparisons test).

In order to be therapeutically useful, the newly induced vasculature needs to: a) persist in the long-term, to ensure efficacy, and b) avoid progressive uncontrolled growth, even of morphologically normal capillary networks, to ensure safety. We therefore studied the long-term fate of the newly formed vasculature induced by VEGF and PDGF-BB co-expression up to 4 months after cell implantation, both in the ear and leg muscles. Control cells fused into muscle fibers and still persisted after 4 months, as shown by X-gal staining, while the morphology of pre-existing vasculature was not altered (Fig. 6a,b). Since expression of PDGF-BB alone did not induce any angiogenesis by 4 weeks at any dose, this group was not repeated. The normal capillary networks induced by V-low and V-med clones persisted unchanged (Fig. 6c,d), whereas high VEGF levels alone (V-high) caused the aberrant enlarged structures induced by 4 weeks to develop into large macroscopic cavernous angiomas already after 6 and 9 weeks in the ear and leg muscles respectively, which required early termination of the experiment for this group (Fig. 6e). Co-expression of PDGF-BB with low and medium VEGF levels did not modify the normal morphology of newly induced capillaries compared to the 4 week time-point (Fig. 6f,g). The normalized capillary networks induced by high-level co-expression (VIP-high) also remained stable and did not progress towards angioma-like structures (Fig. 6h). The quantification of vessel length density showed that, in all conditions leading to morphologically normal capillaries (V-low, V-med, VIP-low, VIP-med and VIP-high), induced vessels neither regressed nor increased in quantity over 4 months, despite continued expression of the transgenes, as determined by the persistence of the β-gal marker production (blue fibers in Fig. 6). Rather, vessel quantities were stable compared to the 4-week time point (Fig. 3a), similar in all conditions and about double compared with controls (V-low = 119.8 ± 2.6 mm/mm^2^; V-med = 116.1 ± 1.5 mm/mm^2^; VIP-low = 117.2 ± 2.6 mm/mm^2^; VIP-med = 120.4 ± 2.2 mm/mm^2^; VIP-high = 123.6 ± 2.4 mm/mm^2^; Controls = 66.6 ± 2.0 mm/mm^2^; Fig. 6i).

**Figure 7:**
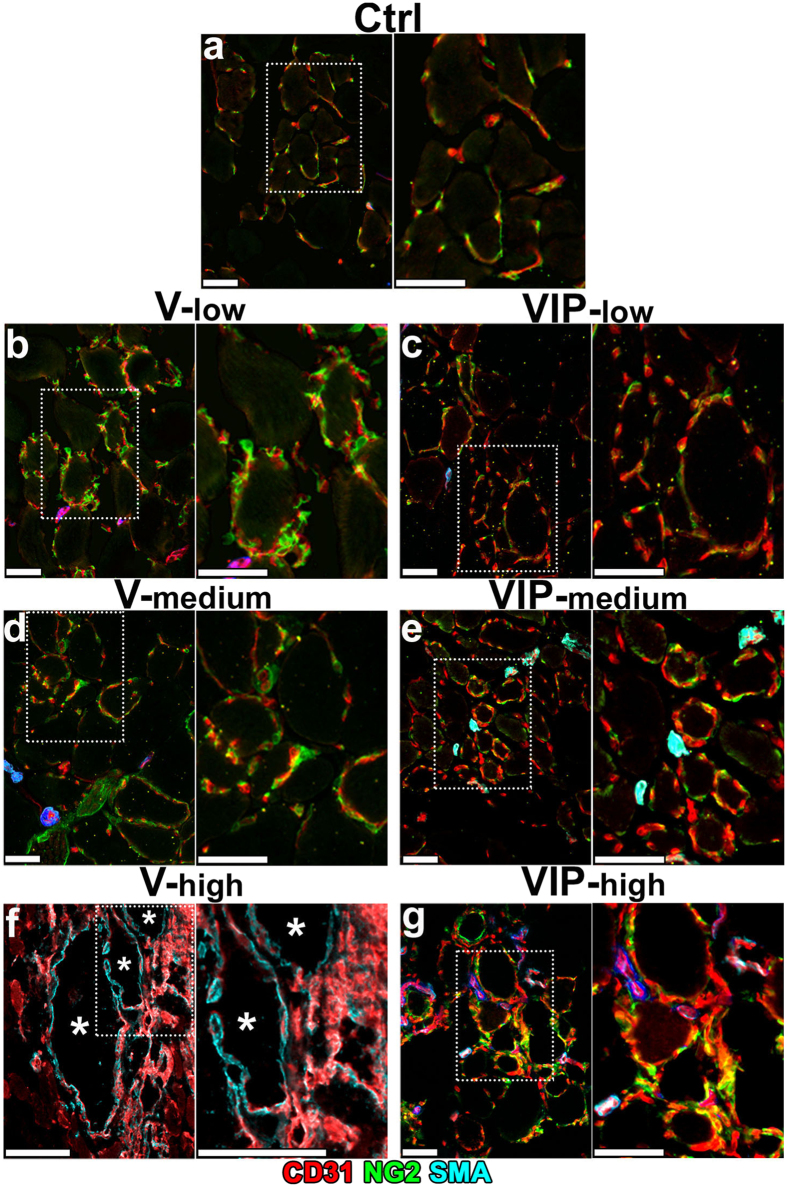
Maturation of capillaries induced by long-term co-expression of VEGF and PDGF-BB. V and VIP myoblasts clones, expressing low, medium or high levels, as well as control cells (Ctrl), were implanted into calf muscles of SCID mice. Frozen sections were obtained 4 months after implantation and immunostained for CD31 (endothelium, in red), NG2 (pericytes, in green) and α-SMA (smooth muscle cells, in cyan); n = 3–7 samples/group. In each panel, the right image is a higher-magnification view of the area marked by the white dotted box in the left image. High VEGF levels caused the progressive growth of macroscopic angiomas (**f**) and required early termination of the experiment after 9 weeks (n = 4). Size bars = 20 μm, except in (**f**): size bar = 100 μm; asterisks in (**f**) indicate large angioma-like aberrant vascular structures.

**Figure 8:**
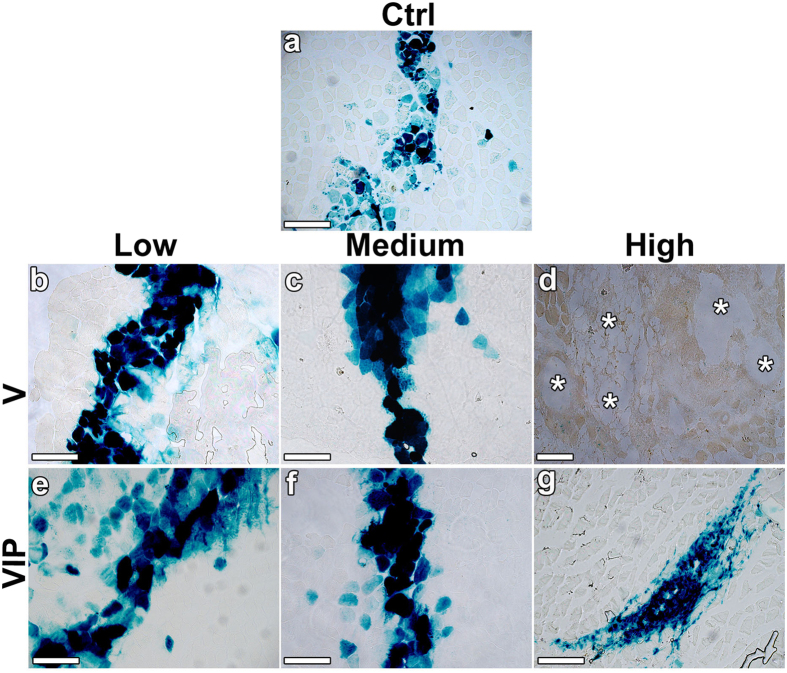
Long-term myoblast engraftment. Myoblast engraftment was revealed by X-Gal staining (blue) on frozen sections of the same muscles analyzed in Fig. 7. Cell engraftment occurred only along the needle tracks of cell injections and all the conditions showed similar engraftment, except for the high VEGF condition (**d**), in which no myoblasts survived (n = 3–7 samples/group). Size bars = 200 μm, except in (**d**): size bar = 400 μm; asterisks in (**d**) indicate large angioma-like aberrant vascular structures.

The long-term maturation of induced vasculature was analysed by immunostaining on frozen sections of leg muscles 4 months after cell implantation. All morphologically normal capillary networks induced by V-low, V-med, VIP-low, VIP-med and VIP-high conditions were uniformly associated with NG2-positive pericytes (Fig. 7b–e,g), similarly to the normal pre-existing capillaries in muscles implanted with control cells (Fig. 7a). The large angiomas induced by V-high cells lacked pericytes, but were covered by a thick smooth muscle coat, as expected (Fig. 7f). Remarkably, no accumulation or expansion of NG2-positive cells, beyond the individual pericytes associated with vessels, was observed in any of the muscles, even in the PDGF-BB-overexpressing conditions (Fig. 7c,e,g). X-gal staining showed that implanted myoblasts stably engrafted along the needle tracks in muscles 4 months after injection (Fig. 8). In all conditions that induced normal angiogenesis (V-low, V-med, VIP-low, VIP-med and VIP-high) and in control tissues myoblast engraftment was similar, consisting both of fusion to pre-existing large fibers and of differentiation into smaller-caliber *de novo* fibers (Fig. 8a–c,e–g). Consistently with previous observations^10^ and with the results in the ear muscles after 4 weeks (Fig. 2f), no myoblasts survived in the V-high condition after 9 weeks (Fig. 8d).

### Dose-dependent arteriole formation

**Figure 9:**
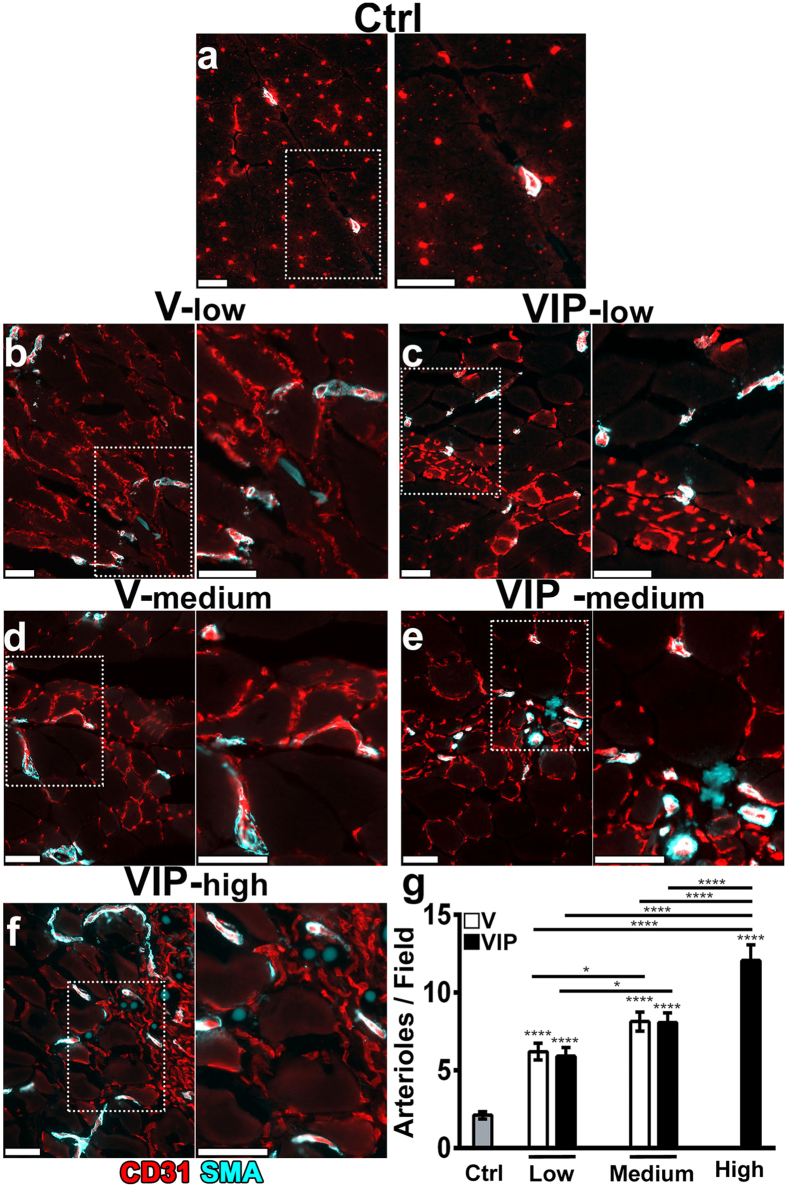
Arteriole formation adjacent to areas of angiogenesis. Capillaries and arterioles were identified by immunostaining on frozen sections of the same muscles analyzed in Figs 7 and 8, 4 months after implantation of control cells (Ctrl) or V and VIP myoblasts clones, expressing low, medium or high levels. (**a–f**) Endothelium is stained in red (CD31) and smooth muscle cells in cyan (α-SMA). In each panel, the right image is a higher-magnification view of the area marked by the white dotted box in the left image. Size bars = 50 μm in all panels. (**g**) Quantification of the number of arterioles adjacent to the areas of increased angiogenesis, which were strictly localized to the sites of myoblast engraftment. Data are shown as mean  ±  SEM (n = 14 measurements/group in 2 tissues/group); ****p < 0.0001 vs control and for indicated comparisons; *p < 0.05 for indicated comparisons (ANOVA with Sidak multiple comparisons test).

Functional vascular networks comprise both microvascular capillaries, responsible for nutrient and respiratory gases exchange, and larger caliber arterioles, capable of supplying increased blood flow and responsible for regulating perfusion of downstream capillaries depending on the metabolic needs of the tissue. Therefore, we identified arterioles in implanted muscles 4 months after myoblast injection as regularly shaped vessels, moderately larger than capillaries (15–30 μm) and covered by a homogeneous smooth-muscle coat^17^. While the angiogenic response to VEGF and PDGF-BB delivery was tightly localized around the expressing myoblasts, a large number of arterioles could be identified exclusively in the zone of muscle fibers immediately adjacent to it (Fig. 9), as previously described with uncontrolled levels of VEGF alone^17^. All conditions that induced normal angiogenesis (V-low, V-med, VIP-low, VIP-med and VIP-high) also exhibited a significantly increased arteriole density in the peri-angiogenic areas compared to control muscles (Fig. 9g). Interestingly, the amount of induced arterioles was proportional to the VEGF dose, while co-expression of PDGF-BB did not affect their density further (Fig. 9g). Notably, the most abundant arteriole induction was observed in the VIP-high condition, reaching about double the density as in the V-low and VIP-low muscles. These results suggest that co-expression of VEGF and PDGF-BB induces physiologically organized vascular trees composed of both arterial and capillary compartments. Further, while PDGF-BB did not modify the VEGF dose-dependent generation of arterioles, it enabled the positive effect of higher VEGF doses by preventing angioma formation.

## Discussion

PDGF-BB co-expression is an attractive strategy to avoid side effects of uncontrolled VEGF delivery, with the goal of improving safety and efficacy of therapeutic angiogenesis. However, long-term expression, which may be necessary to ensure stabilization and persistence of induced angiogenesis, raises safety concerns. Here we found that long-term co-expression of VEGF and PDGF-BB ensures completely normal and stable angiogenesis in the therapeutic target tissue of skeletal muscle. PDGF-BB specifically prevented the appearance of aberrant angioma-like structures with high VEGF doses, instead yielding normal networks of mature capillaries, but did not affect the already normal angiogenesis induced by low and medium VEGF doses.

Both VEGF and PDGF-BB exhibit strong binding to extracellular matrix and exquisite localization in the microenvironment around each producing cell *in vivo*^9^,^18^. This binding leads to the formation of microenvironmental gradients of concentration, which are actually required for the biological function of both factors. In fact, seminal studies in transgenic mice that produce only one of the 3 major splice isoforms of VEGF, which differ in their affinity for extracellular matrix and therefore in their degree of diffusion and the shape of formed gradients, showed that balanced matrix binding is required to ensure proper vessel morphogenesis; whereas too little or too much of it resulted in dysfunctional vascular networks, displaying excessive enlargement or hyperbranching, respectively^19^. On the other hand, deletion of the matrix-retention motif from the endogenous *Pdgfb* gene, leading to free diffusion of the expressed protein, paradoxically caused detachment of pericytes from endothelium rather than their recruitment, and resulted in defective angiogenesis^20^.

Direct gene transfer, such as with clinically employed viral vectors, leads to heterogeneous expression levels in the tissue and it is unsuitable to study the effects of specific factor doses localized in the microenvironment. Therefore, in order to rigorously investigate the dose-dependent effects of VEGF and PDGF-BB co-expression we took advantage of a highly controlled gene delivery platform, based on monoclonal populations of transduced myoblasts^10^: since all cells in the implanted monoclonal populations share the same copy number and genomic integration sites of the retroviral vectors, the global effects in the muscles reflect the actions of a specific microenvironmental dose of each factor. Furthermore, we previously showed that the co-expression of VEGF and PDGF-BB from a single bicistronic vector ensures that both factors generate overlapping gradients around producing cells in the tissue at balanced levels^12^. This is a key aspect to ensure consistent pericyte recruitment to the activated endothelial structures, as co-expression of PDGF-BB and VEGF from separate viral vectors led instead to their detachment from nascent vessels^21^.

While the normal angiogenesis induced by V-low and V-medium levels was not affected by PDGF-BB, the VIP-high condition a) restored vessel length density to the same level as lower VEGF doses, and b) yielded capillary networks with diameters similar to the V-med and larger than V-low (Fig. 3b, p < 0.01). These results bear therapeutic relevance. In fact, using the same delivery platform we previously found that effective blood flow improvement in a hindlimb ischemia model required a combined increase in vessel number and size. Only the V-med clones effectively provided this optimal functional outcome, whereas similar amounts of equally normal, but smaller, vessels induced by the V-low clones could not improve tissue perfusion^11^. Therefore, PDGF-BB co-expression not only prevented the growth of angioma-like structures by high VEGF doses, but also yielded vascular networks with the morphometric characteristics required for efficacious functional improvement. In this respect, it should be noticed that the robust growth of normal microvascular capillary networks was also accompanied by the generation of numerous arterioles in the immediately adjacent areas. In fact, while increased capillary density can facilitate metabolic exchanges, functional improvement requires the generation of new arterial vessels (arteriogenesis) to supply increased blood flow to the affected tissues^22^. Interestingly, we found that the amount of induced arterioles was directly dependent on VEGF dose. VEGF is known to favor arterial over venous differentiation and to be required for adult arteriogenesis^23^, but to our knowledge this is the first report showing that the arteriogenic effect of VEGF in adult skeletal muscle is dose-dependent. PDGF-BB did not show any direct arteriogenic function with low and medium VEGF levels. However, by preventing the growth of aberrant angioma-like vascular structures, PDGF-BB co-delivery with high doses of VEGF enabled the highest increase in arteriole density of all conditions, i.e. about double compared to V-low/VIP-low and six times the control values. These data lend further support to the concept that the therapeutic potential of VEGF may be expanded by increasing the safety of higher doses through PDGF-BB co-delivery. In agreement with these observations, co-expression of VEGF and PDGF-BB at uncontrolled levels has been previously found to significantly improve tissue perfusion in ischemic hind-limbs compared to VEGF alone^12^.

It is interesting to note that the results in Figs 3 and 6 show how all conditions that lead to normal angiogenesis, whether by VEGF alone or together with PDGF-BB co-expression, induced a remarkably similar increase in vessel length density of slightly less than 2-fold compared to controls. A possible explanation for this similarity in the amount of induced angiogenesis, despite very different factor doses, may be provided by our recent finding that VEGF over-expression in skeletal muscle, at the doses required for functional benefit, does not cause vascular growth by the well-known process of sprouting, but rather through an initial circumferential enlargement of pre-existing vessels followed by longitudinal splitting into two new capillaries, i.e. intussusceptive angiogenesis^16^. As this process essentially leads to a stereotyped doubling of vessels in affected areas, it is tempting to speculate that PDGF-BB co-expression may normalize VEGF-induced aberrant angiogenesis by regulation of intussusceptive vascular growth. This hypothesis remains to be investigated by *ad hoc* experiments.

A particularly relevant finding from a clinical point of view is that balanced co-expression of VEGF and PDGF-BB from a single vector ensured the long-term safety and stability of induced angiogenesis. Notably, not only did the balanced co-expression prevent the appearance of aberrant angiogenesis by 4 weeks, but also stably avoided the emergence of even microscopic angioma-like structures for up to 4 months, despite the delivery of high VEGF levels. This is in sharp contrast to the induction of aberrant vessels by similar levels of VEGF alone within 4 weeks, which progressively grew into macroscopic and life-threatening hemangiomas already by 6 weeks, requiring termination of the experiment. It is interesting to note that aberrant structures induced by high VEGF alone continued growing into macroscopic angiomas between 4 and 9 weeks despite disappearance of the V-high myoblasts (Fig. 8d), in agreement with previous observations with both clonal and polyclonal populations of VEGF-expressing myoblasts^10^, indicating that high VEGF levels are only required in the early stages of aberrant angiogenesis, which then becomes a self-sustaining process. The underlying mechanism remains to be fully elucidated. However, blockade experiments indicate that sustained angioma growth still depends on VEGF signalling, likely from endogenous sources. In fact, in experiments with the same myoblast-based delivery platform, VEGF sequestration by treatment with the receptor-body Aflibercept (VEGF-Trap) caused aberrant angiogenesis to regress both after 2, 3 and 4 weeks, when implanted V-high myoblasts had already disappeared and could no longer provide VEGF production, whereas normal angiogenesis induced by lower VEGF levels had completely stabilized by 4 weeks and could persist despite VEGF blockade^10^,^24^.

The use of monoclonal myoblast populations, in which every cell produces the same levels of VEGF and PDGF-BB, excludes the possibility that the observed phenotypic conversion from angioma-like vascular structures to normal capillary networks could be due to a selection of subpopulations producing optimal VEGF levels. Furthermore, the analysis of *in vivo* expression levels also excludes the possibility that the angiogenic normalization could be due to a down-regulation of VEGF expression by PDGF-BB, as the transcripts for both exogenous (retroviral vector-encoded) and endogenous VEGF were similar or higher in the tissues implanted with VIP clones compared to the corresponding V clones. The analysis was focused on the first week after implantation because it has been previously determined that the transition between normal and aberrant angiogenesis by increasing VEGF doses crucially takes place during the initial morphogenic events in a well-defined time window between 4 and 7 days after factor delivery^16^.

Therapeutic efficacy depends not only on the efficient induction of normal and functional vascular networks, but also on their long-term persistence. It is therefore important that vessel length density quantifications showed that the amount of induced angiogenesis remained stable between 4 weeks and 4 months, retaining the features of mature vasculature, associated with normal NG2-positive pericytes that established tight cell-to-cell contacts with the endothelium^25^,^26^. On the other hand, stabilization and persistence of newly induced vessels is not exclusively regulated by the PDGF-BB/pericyte axis^27^. For example, Angiopoietin-1, which is normally secreted by pericytes and activates its endothelial receptor Tie2, has been shown to restore the vascular defects caused by pericyte ablation in the retina, stabilizing vessels and preventing edema and hemorrhage despite the persistent absence of mural cells^28^. Also inflammatory cells of different origin significantly contribute to vascular stabilization and persistence, such as pro-angiogenic CXCR4 + myeloid cells^29^ or Neuropilin1-expressing monocytes that produce several vascular maturation factors and can be recruited by endothelial Semaphorin3A^24^,^30^. The regulation of recruitment and function of pro-maturative myeloid populations by PDGF-BB remains to be investigated.

In conclusion this study shows that VEGF and PDGF-BB co-expression by a single bicistronic construct promotes long-lasting and safe angiogenesis in a mouse model, suggesting that this approach may have promising potential to overcome challenges of VEGF gene therapy. It should be underlined that the use of monoclonal populations of transduced myoblasts would not be directly employable in a clinical setting, but rather provides controlled conditions for a proof-of-concept, which should be translated towards a clinical application using other gene delivery platforms, such as AAV vectors. On the other hand, the library of monoclonal populations described here provides a highly standardized platform for future studies to investigate the cellular and molecular mechanisms controlling the switch between normal and aberrant angiogenesis by VEGF dose and its regulation by pericyte recruitment.

## Methods

### Cell culture

Primary myoblasts isolated from C57BL/6 mice and transduced to express the β-galactosidase marker gene (lacZ) from a retroviral promoter^31^ were over-infected at high efficiency^32^ with the retroviruses expressing murine VEGF_164_, human PDGF-BB, or both at a fixed ratio to each other from a bicistronic cassette through the encephalomyocarditis virus Internal Ribosomal Entry Site (IRES) as previously described^12^. A truncated version of CD8a (trCD8a) was co-expressed with VEGF or PDGF-BB from a similar bicistronic cassette (V and P), or from a separate promoter (VIP), as a convenient cell surface marker of transduced cells, as described^15^. Single cells were isolated from the resulting V, P and VIP (Vegf-Ires-Pdgfb) transduced populations by FACS, based on their CD8 staining, using a Vantage SE cell sorter (Becton Dickinson, Basel, Switzerland). Single cell isolation was confirmed visually and monoclonal populations of transduced myoblasts were expanded in culture to express specific and homogeneous levels of each factor, as previously described^10^,^11^,^15^. All myoblast populations were cultured in 5% CO_2_ on collagen-coated dishes, with a growth medium consisting of 40% F10, 40% DMEM low glucose (1000 mg glucose/L) (Sigma-Aldrich Chemie GmbH, Steinheim, Germany) and 20% fetal bovine serum (HyClone, Logan, UT) supplemented with 2.5 ng/ml basic fibroblast growth factor (FGF-2) (Becton Dickinson, Basel, Switzerland), as described^33^.

### VEGF_164_ and PDGF-BB ELISA measurements

Cell culture supernatants were quantified for mVEGF_164_ and hPDGF-BB protein using an ELISA kit (R&D Systems Europe, Abingdon, UK). The stability of VEGF and PDGF-BB secretion was assessed periodically during *in vitro* expansion of different cell batches. One ml of medium was harvested from myoblasts cultured in one 60 mm dish, following a 4-hour incubation, filtered and analyzed in duplicate. Medium was supplemented with 10 μg/ml heparin to prevent retention of PDGF-BB on the cell surface. Results were normalized for the number of cells and time of exposure to medium. Four dishes of cells were assayed for each clone, which was used for *in-vivo* experiments (n = 4).

### *In vivo* myoblast implantation

In order to avoid an immunological response to engineered myoblasts that express β-galactosidase and hPDGF-BB, 6–8 week-old immunodeficient SCID CB. 17 mice (Charles River Laboratories, Sulzfeld, Germany) were used. Animals were treated in accordance with the Swiss Federal guidelines for animal welfare, after approval from the Veterinary Office of the Canton of Basel-Stadt (Basel, Switzerland). Myoblasts were dissociated in trypsin and resuspended in PBS with 0.5% BSA. 5 × 10^5^ myoblasts in 5 μl were implanted into the posterior auricular muscle, midway up the dorsal aspect of the external ear, and into the tibialis anterior and gastrocnemius muscles in the calf, using a syringe with a 30-gauge needle.

### Tissue staining

The entire vascular network of the ear could be visualized following intravascular staining with a biotinylated *Lycopersicon esculentum* lectin (Vector Laboratories, Burlingame, CA) that binds the luminal surface of all blood vessels, as previously described^10^. Mice were anesthetized by i.p. injection of Ketamine (100 mg/kg) and Xylazin (10 mg/kg), lectin was injected intravenously (100 μl per mouse of lectin at 1 mg/ml concentration) and 4 minutes later the tissues were fixed by vascular perfusion of 1% paraformaldehyde and 0.5% glutaraldehyde in PBS pH 7.4. Ears were then removed, bisected in the plane of the cartilage, and stained with X-gal staining buffer (1 mg/ml 5-bromo-4-chloro-3-indoyl-β-D-galactoside, 5 mM potassium ferricyanide, 5 mM potassium ferrocyanide, 0.02% Nonidet P-40, 0.01% sodium deoxycholate, 1 mM MgCl_2_ in PBS pH 7.4). Tissues were stained using avidin-biotin complex-diaminobenzidine histochemistry (Vector Laboratories, Burlingame, CA), dehydrated through an alcohol series, cleared with toluene and whole-mounted on glass slides with Permount embedding medium (Fisher Scientific, Wohlen, Switzerland). For tissue sections, mice were anesthetized as above, sacrificed by cervical dislocation and the entire tibialis anterior and gastrocnemius muscles or whole ears were harvested, embedded in OCT compound (Medite, Basel, Switzerland), frozen in freezing isopentane and cryosectioned. Alternatively, mice were anesthetized and the tissues were fixed by vascular perfusion of 1% paraformaldehyde in PBS pH 7.4 for 3 minutes. Entire tibialis anterior and gastrocnemius muscles excised from perfused mice were fixed for additional two hours in 0.5% paraformaldehyde and then immersed overnight in 30% sucrose solution for cryoprotection before being embedded in OCT compound and frozen. Tissue sections were then stained with X-gal (20 μm sections) or with H&E (10 μm sections) as previously described^31^,^32^. Immunofluorescence was performed as previously described^10^. The following primary antibodies and dilutions were used: rat monoclonal anti-mouse PECAM-1 (clone MEC13.3, BD Biosciences, Basel, Switzerland) at 1:100; mouse monoclonal anti-mouse α-SMA (clone 1A4, MP Biomedicals, Basel, Switzerland) at 1:400; rabbit polyclonal anti-NG2 (Chemicon International, Hampshire, UK) at 1:200. Fluorescently labeled secondary antibodies (Invitrogen, Basel, Switzerland) were used at 1:200.

### Vessel measurements

Vessel length density (VLD) and diameters were measured in whole mounts of ears stained with *L. esculentum* lectin as previously described^10^. Briefly, vessel diameters were measured by overlaying a captured microscopic image with a square grid. Squares were chosen at random, and the diameter of each vessel (if any) in the center of selected squares was measured. Two hundred total vessel diameter measurements were obtained from 6
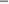
10 ears per each group (n = 6–10). Vessel length density was measured on 1 to 3 fields per ear in 6–10 ears per each group (n = 6–10) by tracing the total length of vessels in the fields and dividing it by the area of the fields. All image measurements were performed with AnalySIS D software (Soft Imaging System, Gmbh, Münster, Germany). The number of arterioles was quantified in fluorescently immunostained cryosections. Briefly, arterioles were defined as vessels of regular shape and moderately larger than capillaries (15–30 μm) associated with a thick and homogeneous smooth muscle layer (positive for α-smooth muscle actin, α-SMA) coating the endothelial layer (positive for CD31), as previously described^17^, and were counted on 14 microscopic fields/group, localized in proximity to the areas of induced angiogenesis (n = 2 muscles/group; field size = 180′710 μm^2^).

### Quantitative Real Time-PCR

Whole fresh mouse muscles were disrupted using a Qiagen Tissue Lyser (Qiagen, Hombrechtikon, Switzerland) to extract total RNA with a commercial kit (Qiagen, Hombrechtikon, Switzerland), according to the manufacturer’s instructions (n = 4 muscles per group). Total RNA was reverse-transcribed into cDNA with the Omniscript Reverse Transcription kit (Qiagen, Hombrechtikon, Switzerland) at 37 °C for 60 minutes. Quantitative Real-Time PCR (qRT-PCR) was performed on an ABI 7300 Real-Time PCR system (Applied Biosystems, Zug, Switzerland). In order to quantify mouse VEGF_164_ transcripts from the transduced myoblasts Specific sets of primer and probe sequences were designed with Primer Express software 3.0 (Applied Biosystems, Zug, Switzerland) to quantify mouse VEGF_164_ transcripts from the exogenous transduced myoblasts (Exo), based on a sequence expressed by the VEGF retroviral construct, or from the endogenous gene (Endo): Exo V-forward: 5′-GCTCTCCTCAAGCGTATTCAACA; Exo V-reverse: 5′-CCCCAGATCAGATCCCATACA; Exo V-probe: 5′-FAM-CTGAAGGATGCCCAGAAGGTACCCCA-TAMRA; Endo V-forward: 5′-GACGGGCCTCCGAAACC; Endo V-reverse: 5′-TGGTGGAGGTACAGCAGTAAAGC; Endo V-probe, 5′-FAM-AACTTTCTGCTCTCTTGGGTGCACTGGAC-TAMRA.

To determine the expression of the other genes the following TaqMan gene expression assays were used from Applied Biosystems (Applied Biosystems, Zug, Switzerland): murine *Pdgfb* (Mm00440678_s1), human *Pdgfb* (Hs00234042_m1) and murine *Gapdh* housekeeping gene (Mm03302249_g1). The cycling parameters were: 50 °C for 2 minutes, followed by 95 °C for 10 minutes and 40 cycles of denaturation at 95 °C for 15 seconds and annealing/extension at 60 °C for 1 minute. All primers were used at 400nM, except the Endo-V probe at 100nM. Reactions were performed in triplicate for each template, averaged, and normalized to expression of the *Gapdh* housekeeping gene.

### Statistics

Data are presented as means ± standard error. The significance of differences was evaluated with the GraphPad Prism 6 software (GraphPad Software). Multiple comparisons were performed using analysis of variance (ANOVA) followed by the Sidak test. Gene expression data representing fold-changes versus control, which are asymmetrically distributed, were first normalized by logarithmic ln(y) transformation and then analyzed by 1-way ANOVA followed by the Sidak test for multiple comparisons; p < 0.05 was considered statistically significant.

## Additional Information

**How to cite this article**: Gianni-Barrera, R. *et al.* Long-term safety and stability of angiogenesis induced by balanced single-vector co-expression of PDGF-BB and VEGF_164_ in skeletal muscle. *Sci. Rep.* **6**, 21546; doi: 10.1038/srep21546 (2016).
